# Supplementary material for: One-Step Preparation of Both Micron and Nanoparticles
Source: Polymers (Basel). 2024 Nov 7;16(22):3120. doi: 10.3390/polym16223120 (PMC11597913; doi:10.3390/polym16223120)
Supplement: Supplementary file 1 [file polymers-16-03120-s001.zip › polymers-3239424-supplementary.pdf]

One-step preparation of both micron and nanoparticles

Zihao Guo,[a] Zhiyuan Zhang,[a] Yunchen Cao,[b] Chunyi Chen,[a] Juan Wang,[a,*] Haoran Yang,[a]

Wenbin Song,[a] Yiyang Peng,[a] Xiaowei Hu[a,*]

a School of Chemistry & Chemical Engineering, Linyi University, Linyi 276000, P. R. China

b Linyi Hongrun Environmental Testing CO.,LTD, Linyi 276000, P. R. China

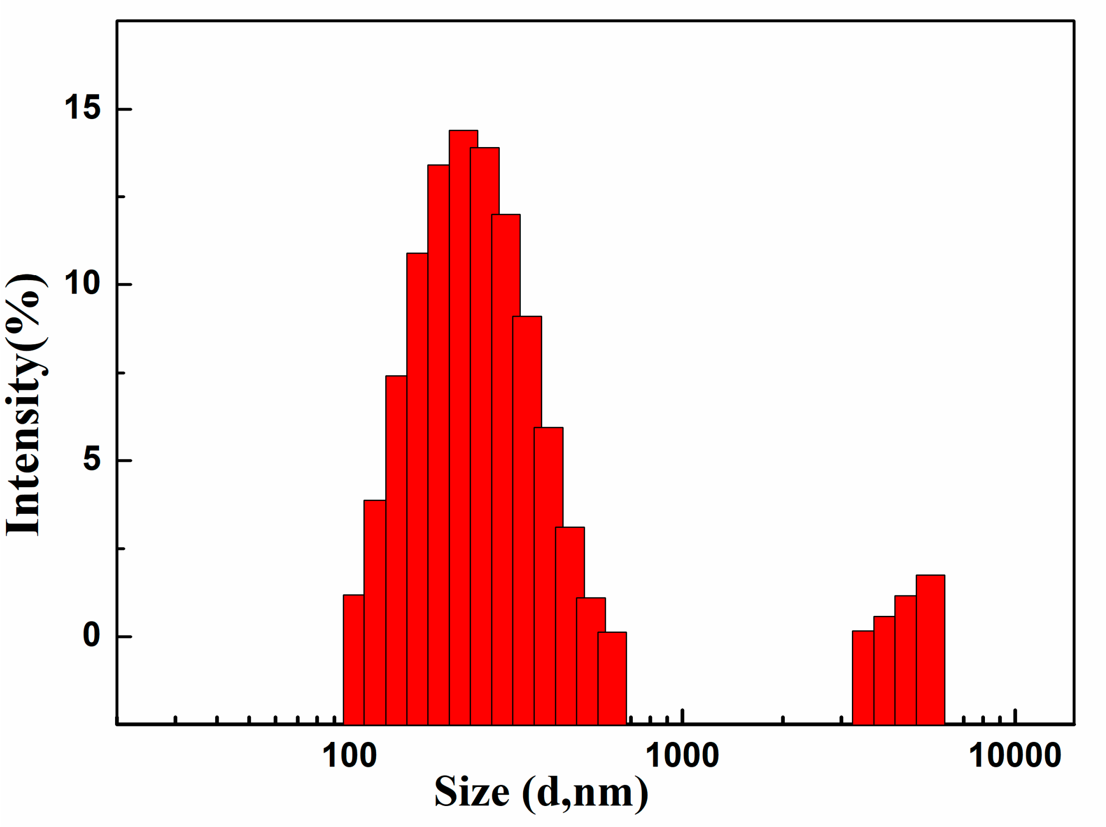

Figure S1. DLS z-average distributions of micron and nano sized particles

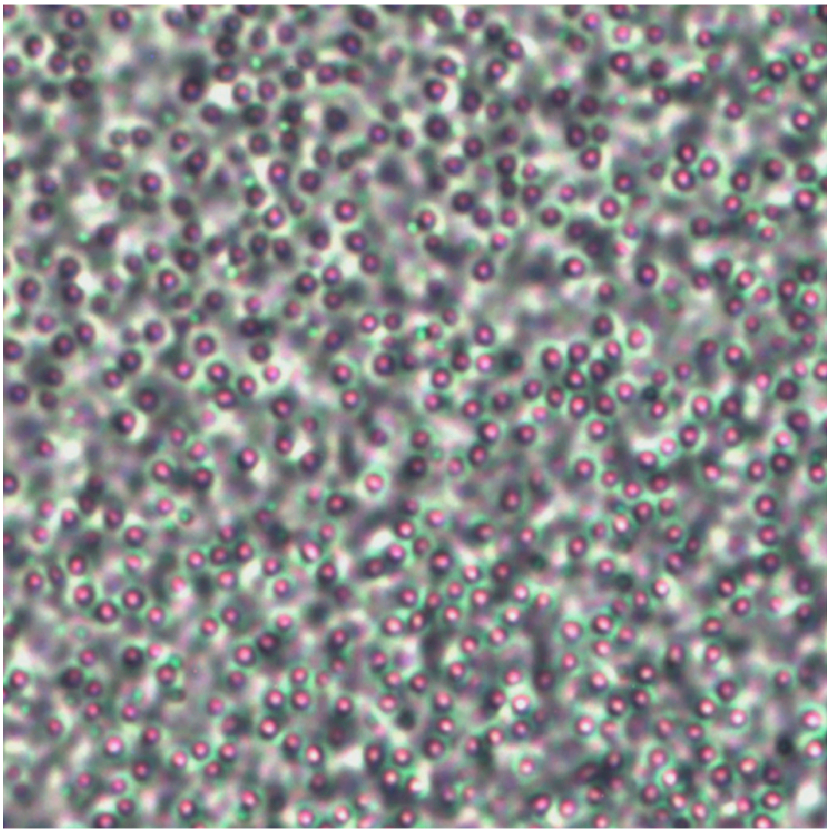

Figure S2 the optical microscope photo of membrane formed by MNPs
